# Reproductive and Economic Efficacy of Three FTAI Protocols in Four Weather Seasons in a High-Yielding Dairy Farm from Romania

**DOI:** 10.3390/vetsci13101042

**Published:** 2026-10-03

**Authors:** Amalia-Ioana Hârbu, Dan Drugociu, Silviu-Ionuț Borș

**Affiliations:** 1 Clinics Department, Faculty of Veterinary Medicine, “Ion Ionescu de la Brad” Iași University of Life Sciences, 700490 Iași, Romania; amalia.harbu@iuls.ro (A.-I.H.); dan.drugociu@iuls.ro (D.D.); 2 Research and Development Station for Cattle Breeding Dancu, 707252 Iaşi, Romania

**Keywords:** conception rate, dairy cows, timed artificial insemination, heat stress, economics

## Abstract

This study evaluates the effectiveness and financial impact of three fixed timed artificial insemination (FTAI) protocols—an intensive Double Ovsynch (DO) protocol, OVS5D, a modified Ovsynch protocol incorporating GnRH administration on day 5 post-FTAI, and a standard Ovsynch (OVS) protocol, beginning with the first artificial insemination (AI) on a high-yielding dairy farm. For this study, three groups were assessed: the OVS group (*n* = 904), the OVS5D group (*n* = 830), and the DO group (*n* = 859). To assess the economic impacts of each protocol, the UW-Cornell DairyRepro$ decision tool was used. The results revealed that the DO group had a significantly higher conception rate (CR) at the first AI (53.08%, *p* < 0.05) compared to both the OVS (47.46%) and OVS5D (46.27%) groups, which showed similar results (*p* > 0.05). At the second AI, the CRs for the DO (45.18%) and OVS (44.97%) groups were comparable (*p* > 0.05), with both groups achieving higher CRs than the OVS5D group (35.67%, *p* < 0.05). Among the three groups, primiparous cows exhibited similar CRs (54.32 vs. 57.77 vs. 55.45, *p* > 0.05), while multiparous cows showed higher CRs (*p* < 0.05) in the DO group (52.64%) compared to the OVS (42.64%) and OVS5D (42.46%) groups. In our study, there is a clear interaction between synchronization protocols, parity, and seasonal variations on high-yielding dairy cows’ CRs. Compared to the OVS group, the net present value (NPV) per cow per year was EUR 35 higher in the DO group and EUR 11.8 lower in the OVS5D group. Seasonal analysis across the four weather seasons (spring, summer, autumn, and winter) revealed that all three protocols showed substantially reduced CRs during summer, confirming universal heat stress susceptibility in standard-cooled herds regardless of synchronization intensity. Based on these findings, the DO protocol is recommended for multiparous cows and standard OVS for primiparous cows; the OVS5D is not recommended due to inferior fertility and negative economic returns. These recommendations are specific to high-yielding Holstein herds managed under temperate continental climate conditions similar to this study farm and should not be over-generalized to other production systems or climates.

## Simple Summary

This study compares the reproductive and economic performance of three fixed timed artificial insemination (FTAI) protocols—standard Ovsynch (OVS, *n* = 904), a modified Ovsynch with GnRH on day 5 post-AI (OVS5D, *n* = 830), and Double Ovsynch (DO, *n* = 859)—across four seasons on a high-yielding dairy farm in Romania (*n* = 2593 cows). The study specifically focuses on determining the optimal reproductive strategies for primiparous and multiparous cows. In this context, using the UW-Cornell DairyRepro$ decision tool, our study showed that the DO protocol is optimal for multiparous cows, delivering higher conception rates (CRs) and the best economic return (EUR +35/cow/year versus OVS), while standard OVS is sufficient for primiparous cows. All three protocols showed reduced CRs during summer regardless of synchronization intensity, and the OVS5D is not recommended due to inferior fertility and a negative economic return (EUR −11.8/cow/year versus OVS).

## 1. Introduction

Recent advancements in the reproductive physiology of dairy cows have led to the development of innovative management strategies and technologies aimed at enhancing reproductive performance on high-yielding commercial dairy farms. These advancements encompass a broad spectrum of approaches, including refined breeding protocols, nutritional management, and monitoring systems that optimize the tracking of reproductive cycles.

To improve profitability, commercial dairy farms continuously evaluate the most effective reproductive technologies, such as artificial insemination (AI) and embryo transfer, alongside electronic estrus detection methods and fixed-timed artificial insemination (FTAI) protocols. Farms must also consider the interplay of cow health, feed quality, and environmental conditions, ensuring these factors support optimal reproductive outcomes. Effective implementation of these strategies requires farm managers to integrate zootechnical knowledge with an understanding of the economic consequences of reproductive decisions. By combining scientific insights with practical management approaches, dairy operations can enhance reproductive efficiency and profitability [1].

A significant challenge for high-yielding dairy farms is the incidence of anovulation, with prevalence ranging from 5% to 45% of dairy cows by the end of the voluntary waiting period (VWP) [2]. Research by Walsh et al. [2], Santos et al. [3], and Bamber et al. [4] underscores how the prevalence of anovulation adversely impacts reproductive performance by increasing the median time to pregnancy and overall productivity. Reproductive performance does not influence milk yield directly. Rather, delayed conception lengthens the calving interval, which increases the proportion of cows in late lactation with lower daily yields and postpones the onset of the subsequent lactation. The economic effect of impaired fertility is therefore expressed through herd-level productivity rather than through the individual lactation curve. Addressing this issue is vital for improving fertility rates and ensuring the sustainability of high-yielding dairy farms through the appropriate application of FTAI protocols. However, the fact that fertility problems can be reduced through high-level management suggests that the antagonism between milk production and fertility is more environmental than genetic [5].

A common reproductive strategy in dairy herds involves submitting cows detected in estrus to AI at the end of the VWP, while other cows are treated with the Ovsynch (OVS) protocol for FTAI [6]. The use of OVS in dairy cows has been shown to reduce the median days to first AI and days open compared to cows receiving AI after estrus detection [7]. Furthermore, cows submitted to AI after OVS achieve conception rates (CRs) similar to those of cows receiving AI after estrus detection (39% vs. 37%) [7] with limited hormone use. However, CRs to FTAI after OVS may be influenced by variable synchronization rates [8,9,10] and the fertility of anovular cows at FTAI [11]. Modifications to the original OVS protocol, such as Double Ovsynch (DO) [12,13] and the application of GnRH post-AI [14,15,16], have demonstrated notable success. These protocols enhance the rate of AI services and significantly boost CRs, resulting in reproductive performance that is unprecedented for high-yielding Holstein dairy cows [12].

While primiparous and multiparous cows may benefit from tailored reproductive strategies that allow for individualized VWPs [17], many modern dairy farms adopt a uniform reproductive program for simplicity and management efficiency. The timing of AI, combined with precise control over follicular development and ovulation using various FTAI protocols, can help minimize the effects of sperm longevity. Even bulls with a short sperm lifespan can effectively cover the ovulation window, leading to acceptable CRs [18]. This approach allows farmers to streamline operations while maintaining effective reproductive management across the entire herd [19].

Although the OVS protocol has become the dairy industry standard for estrus synchronization and subsequent AI, it has been associated with limitations, particularly regarding low CRs in high-yielding dairy cows [12,20]. Consequently, these limitations have prompted researchers to develop innovative strategies aimed at improving pregnancy rates in cows subjected to FTAI protocols.

One such strategy involves the addition of a supplementary GnRH dose on day 5 after the first FTAI protocol. This intervention is intended to enhance luteal function, potentially reducing embryonic loss during the early stages of gestation [21]. However, the application of this therapeutic approach remains controversial, as questions persist regarding the optimal management of hormonal treatments and their effects on fertility outcomes [20].

In response to these challenges, researchers have recommended the DO protocol, which consists of an initial OVS protocol followed by a consecutive OVS application [20,22]. This sequential approach aims to maximize estrus synchronization, potentially improving reproductive performance in high-yielding dairy cows, as demonstrated by Souza et al. [22] and Wiltbank et al. [23].

While numerous studies have assessed CRs and time to conception through various reproductive programs [7,24,25], there are only a few that have examined the economic advantages of implementing these programs [13,15].

To assess the overall economic viability of various FTAI programs, simulations incorporating reproductive performance alongside associated costs and benefits can be implemented on a farm-specific basis. Previous research by Cabrera [26] and Cabrera and Giordano [27] emphasizes the necessity for tailored strategies that accommodate the unique characteristics of each farm. Additional studies by Giordano et al. [28,29], Kalantari and Cabrera [30], and Borș et al. [13,15,31,32] highlight how such simulations can offer detailed insights into reproductive outcomes, enabling data-driven decision-making based on specific parameters. By evaluating expected net returns, as demonstrated by De Vries et al. [33], Fricke et al. [34], and Cabrera [35], stakeholders can quantify the economic implications of various reproductive management strategies. Understanding these economic implications is therefore essential for optimizing reproductive management and maximizing profitability in high-yielding dairy farms.

Currently, there is a lack of studies with a large sample size assessing the economic outcomes of the modified OVS5D and DO protocols, particularly within high-yielding dairy farms located in temperate continental climates [15,16,32]. Specifically, three key gaps remain unaddressed: (1) the lack of season-stratified fertility data comparing these protocols across all four weather seasons within a single herd; (2) the absence of economic comparisons stratified by parity, despite known differences in postpartum reproductive physiology between primiparous and multiparous cows; and (3) the scarcity of economic parameter calibrations specific to European, and particularly Romanian, high-yielding dairy production systems, as most existing decision-support tools rely on North American cost structures. While various studies have evaluated outcomes associated with these protocols, the debate regarding which protocol is recommended remains contentious. As noted by Sales et al. [36], differing results and interpretations contribute to the ongoing discussion, reflecting the complexity of evaluating protocol effectiveness. This gap in research emphasizes the need for an in-depth assessment of the cost-effectiveness, potential financial benefits, and overall economic impact of these alternative approaches. By closely examining the economics of these protocols, this study provides dairy producers with evidence-based insights to enhance reproductive efficiency and overall farm profitability.

To our knowledge, no study has assessed the economic impact of the field-tested OVS protocol compared to alternative reproductive strategies, such as combining OVS with GnRH administration on day 5 post-TAI (referred to as OVS5D) or the DO protocol. The only evaluation available used a deterministic model [28]. Thus, in this study, the UW-Cornell DairyRepro$ decision tool [29] was employed to compare the economic impacts of the widely used OVS protocol against alternative reproductive strategies, such as OVS5D, and the DO protocols. The economic analysis focused on managing reproduction on a high-yield dairy farm in Romania under four distinct seasonal conditions. Unlike our prior single-protocol studies in comparable Romanian herds [13,15,16,31], this is the first study to directly compare OVS, OVS5D, and DO within the same herd, stratified by parity and season, within a unified economic model. This investigation aimed to test three hypotheses: (1) that CR in high-yielding dairy cows can be significantly improved by implementing the OVS5D or DO protocol relative to the standard OVS protocol; (2) that an intensive FTAI protocol, such as DO, could maintain CRs during the summer months at levels comparable to those observed in other seasons; and (3) that overall economic outcomes on high-yielding dairy farms could be improved by implementing either a modified OVS protocol with a GnRH agonist administered on day 5 post-AI, or the DO protocol.

## 2. Materials and Methods

### 2.1. Ethical Approval

All dairy cows were handled in accordance with the directives of the European Union on the protection of animals used for scientific purposes (Dir 2010/63/EU). Ethical review and approval were waived for this study as the study posed no more than minimal risk to the dairy cows. Efforts were made to minimize animal handling and stress.

### 2.2. Animals and Management

All cows enrolled in this study belonged to the same commercial dairy farm with high-yielding Holstein-Friesian cows in northeastern Romania, averaging a milk yield of approximately 14,000 kg over 305 days. During the study period, cows were allocated to one of the three protocols in a balanced sequential manner, based on farm management criteria, as each cow reached the end of the voluntary waiting period (around 60–70 DIM). No formal randomization procedure was applied. Group balance across baseline physiological and management parameters (parity, DIM, milk yield, and BCS) was evaluated and confirmed (*p* > 0.05). Because the proportion of primiparous cows differed among groups (42.4%, 24.8%, and 23.5% in OVS, OVS5D, and DO, respectively), fertility outcomes are reported separately by parity to eliminate parity-related confounding bias. Days-open cows were subjected to the OVS protocol. Calving dates, breeding dates, and DIM were obtained from the AfiMilk management software (version 5.6.3, AfiMilk, Kibbutz Afikim, Israel). During the study period, the VWP was set at around 90 days. The average parity was 1.8 ± 0.9, with a range from 1 to 6 lactations. DIM at the time of first AI averaged 90 ± 8 days, with a range of 88 to 103 days. The average daily milk yield was 47.7 ± 6.3 kg. Additionally, the average body condition score (BCS) on the day of AI was 3.3 ± 0.2, with values ranging from 3.0 to 3.5. All values are presented as mean ± standard deviation (SD). Body condition was assessed using a scale of 1 to 5, developed by Wildman et al. [37] and Edmonson et al. [38]. The scale is defined as follows: 1 = emaciated, 2 = thin, 3 = average, 4 = fat, and 5 = obese. All animals were evaluated by the same veterinarian to ensure consistency in scoring.

The cows were housed in free-stall barns (around 400 cows for each barn) with concrete floors and straw bedding. They were fed a Total Mixed Ration in accordance with the INRA system [39], twice daily, and had unrestricted access to water, adjusted according to their milk production and body size. To maintain the cows’ health, standard management practices were implemented to maintain herd health, including a cooling system connected to a weather station (CMPimpianti, Calvisano, BS, Italy) to mitigate heat stress. During the studied period, the average temperatures were 10.2 °C in spring, 21.3 °C in summer, 11.1 °C in autumn, and 0.8 °C in winter.

Each barn was equipped with a Temperature-Humidity Index (THI) controller, which was also present in the milking area. This system was linked to a central unit that managed fans and water sprayers. The THI was automatically calculated using the following formula: THI = (1.8 × AT + 32) − [(0.55 − 0.0055 × RH) × (1.8 × AT − 26)], where AT represents ambient temperature (°C), and RH represents relative humidity (%) [40].

The cooling system automatically activated when the temperature-humidity index (THI) reached 65, initially operating at low speeds. Sprinkling frequency and duration: Once activated, the sprinklers operated on a 5-min cycle, which included 1 min of wetting followed by 4 min of drying and fan operation. Fan operating hours: The fans ran continuously, 24/7, whenever the THI remained above 65, and turned off when it fell below this threshold. Seasonal dynamics: Cooling was only active during late spring, summer, and early autumn. No cooling was required or applied during late autumn and winter, as THI levels consistently remained below the activation threshold.

The cows were milked three times daily, at 04:00, 12:00, and 19:00, producing an average of around 47 kg of milk per cow per day.

Estrus in cows was identified using the daily estrus report generated by the AfiMilk system (version 5.6.3, AfiMilk, Kibbutz Afikim, Israel). Each identified cow was examined by an experienced veterinary team. Signs indicative of estrus included attempts to mount other cows, chasing herd mates, restlessness, chin resting, sniffing of other cows’ vulvas, bellowing, vaginal congestion, relaxation, and mucus discharge from the vulva. Additionally, standing immobility while being mounted was considered a definitive sign of estrus.

### 2.3. Study Groups

Around the end of the VWP, approximately at 60–70 DIM, cows were allocated in a balanced sequential manner to one of the three study protocols for their first postpartum TAI—OVS, OVS5D, or DO—while days-open cows were subjected to the OVS protocol. The three protocols were applied concurrently throughout the study period, ensuring that all three protocols were represented across all four seasons.

The OVS group (*n* = 904, 383 primiparous and 521 multiparous) was initiated around day 70 of the VWP. Each cow received GnRH (dephereline: gonadorelin acetate [6-DPhe]; Gonavet Veyx, Veyx-Pharma GmbH, Schwarzenborn, Germany) on Day 0, PGF2α (cloprostenol; PGF Veyx, Veyx-Pharma GmbH, Schwarzenborn, Germany) on Days 7 and 8, GnRH on Day 9, and AI 16 h after the final GnRH treatment. The OVS5D group (*n* = 830, 206 primiparous and 624 multiparous) was also initiated around day 70 of the VWP. Each cow received the same reproductive program as the OVS group from the first service, with the addition of a single dose of GnRH on Day 5 post-AI to induce ovulation and accessory corpus luteum (aCL) formation. Finally, cows in the DO group (*n* = 859, 202 primiparous and 657 multiparous) were initiated around day 60 of the VWP with a pre-synchronization phase consisting of GnRH (G1) on Day 0, PGF2α (PG1) on Day 7, and GnRH (G2) on Day 10 (3 days after PG1). This was followed 7 days later (Day 17) by the standard OVS protocol: GnRH on Day 17, PGF2α on Days 24 and 25, GnRH on Day 26, and AI 16 h after the final GnRH injection (Figure 1). Semen from the same bulls was used across all three protocol groups throughout the study period, and sire effect was included as a covariate in the statistical model to account for residual variation in CRs attributable to individual bull differences.

The veterinary team (*n* = 5) was responsible for the reproductive management of the herd. All ultrasound examinations were performed by the lead veterinarian using a 5–7.5 MHz rectal convex probe (BCF EasyScan, BCF Ultrasound Australasia, Mitcham, Victoria, Australia), so that all reproductive assessments were made by a single operator. The remaining four veterinarians administered the hormonal treatments and carried out the inseminations. The reproductive tract was scanned to monitor the reproductive status, confirm pregnancy, and record the presence of an aCL and follicular activity. The criteria for confirming pregnancy included the visualization of an anechoic, fluid-filled uterine horn, indicating the presence of a developing embryo, as well as the identification of a corpus luteum (CL) situated on the ipsilateral side of the uterus. Both ovaries were simultaneously evaluated to identify aCLs. The initial pregnancy diagnosis was performed via transrectal ultrasonography on day 30 post AI. This preliminary assessment was subsequently confirmed on day 90, ensuring accurate tracking of the reproductive cycle. Cows determined to be non-pregnant on day 30 after each AI, were categorized into a days-open group and subjected to the OVS protocol, which is commonly used in days-open cows to synchronize follicular waves and induce ovulation. AIs were conducted 16 h after the second GnRH injection in days open cows and 12 h after the onset of estrus in heat-bred cows. Furthermore, data collected from the pregnancy diagnosis were integrated into the AfiMilk management software (version 5.6.3, AfiMilk, Kibbutz Afikim, Israel), which was also used to evaluate CRs, enabling the veterinary team and farm management to monitor reproductive performance and make informed decisions regarding breeding strategies.

### 2.4. Economic Analysis

A total of 2593 high-yielding dairy cows from a northeastern Romania farm, producing an average of around 14,000 kg of milk per cow over 305 days, were evaluated using the UW-DairyRepro$ decision support tool [28]. The economic support tool included modifications as described by Giordano et al. [29] to assess the economic impact of the OVS protocol as the current first postpartum FTAI protocol compared with the OVS5D and DO protocols. The UW-DairyRepro$ decision support tool assesses reproductive programs, considering the 1st and 2nd AIs.

The NPV of studied reproductive programs during a lactation is defined as the sum of two components: the discounted expected monetary value of the proportion of cows becoming pregnant in consecutive reproductive services until a defined DIM, and the expected monetary value of that proportion of cows not becoming pregnant until the defined DIM [29]. The decision tool integrates a daily herd Markov-chain model that simulates daily state transitions based on reproductive status, parity, and DIM to determine long-term herd economic outcomes.

For this specific commercial herd economic model, the following biological and financial baseline parameters were embedded: average body weight (800 kg), involuntary culling rate (15.5%), mortality rate (4%), stillbirth rate (8%), milk price (EUR 0.50/kg), lactation feed cost (EUR 0.15/kg dry matter [DM]), dry period fixed cost (EUR 0.1/kg DM). Newborn values were set at EUR 200 for female calves and EUR 100 for male calves, while heifer replacement value was EUR 2000, with a residual or salvage value of EUR 1/kg when a dairy cow is culled.

Operational thresholds and reproductive performance data included a VWP of around 90 days across all three groups, a maximum breeding threshold of 200 DIM, a do-not-breed minimum milk yield of 30 kg/day, and gestation lengths of 30 and 90 days at the first and second pregnancy checks, respectively. Protocol-specific inputs derived directly from the experimental data included pregnancy losses (5.5% for OVS, 6.1% for OVS5D, and 6.8% for DO), average days open (116 days for OVS, 134 for OVS5D, and 120 days for DO), CR at first FTAI (47.46% for OVS, 46.27% for OVS5D, and 53.08% for DO), and CR at second FTAI (44.97% for OVS, 35.67% for OVS5D, and 45.18% for DO).

Itemized costs for the reproductive programs consisted of insemination (semen: EUR 20/cow; labor: EUR 5/cow), ultrasound pregnancy checks (EUR 100/h), synchronization injection labor (EUR 15/h), and individual hormone doses (GnRH: EUR 5.00/dose; PGF2α: EUR 2.60/dose). The costs of the OVS, OVS5D, and DO protocols were fully taken into account for the studied cows. The economic decision tool aggregated these variables to calculate the absolute variations in annualized NPV (USD/cow/year) among the reproductive programs, reflecting the specific economic consequences of improvements or reductions in CRs compared with the OVS baseline group. All input costs were converted from USD to EUR prior to analysis. The exchange rate applied was USD 1.00 = EUR 0.81, (National Bank of Romania—BNR—reference rate).

To assess the robustness of the economic outcomes to key cost assumptions, a one-way sensitivity analysis was performed by varying milk price and hormone dose price (GnRH and PGF2α) by ±10% and ±20% around their baseline values, while holding all other model inputs constant.

### 2.5. Statistical Analysis

For statistical analyses, cow parity was classified as either primiparous or multiparous. Cows were categorized based on parity, season, group, and number of AIs. The calving season was classified into four categories: spring (March–May), summer (June–August), autumn (September–November), and winter (December–February). Spring was chosen as the reference season due to its favorable weather conditions. Statistical analyses were conducted using SAS software (ver. 9.4; SAS Institute, Cary, NC, USA). Continuous baseline variables (parity, DIM, milk yield, and BCS) were compared between each alternative protocol (OVS5D, DO) and the reference protocol (OVS) using two-group *t*-tests. Binary logistic regression was used to examine the effect of the FTAI protocol on CR, aCL formation, pregnancy loss, and culling rate, accounting for confounding factors including parity, days in milk, milk yield, days open, sire, and technician effects. A backward elimination procedure was used to retain only statistically significant predictors and their interactions in the final models, following the approach outlined by Hosmer and Lemeshow [41]. Sire and technician effects were tested for inclusion but did not reach statistical significance and were therefore excluded during this process, leaving FTAI protocol (and any significant interactions) as the retained predictor(s) in the final models. Odds ratios (OR) and 95% confidence intervals (CI) were derived from model estimates using Wald limits. Results are presented as proportions, means ± standard deviation (SD), or ORs with their respective 95% CIs, as appropriate. A *p*-value of < 0.05 was considered statistically significant throughout.

## 3. Results

According to the data presented in Table 1, there were no statistically significant differences (*p* > 0.05) in the independent variables among the three groups.

Analysis of the dependent variable (Table 1) revealed noteworthy differences among the three groups (OVS, OVS5D, and DO) across various CR outcomes. Notably, the DO group exhibited a significantly higher CR at the first AI (*p* < 0.05), compared to both the OVS and OVS5D groups, which showed similar results (*p* > 0.05). At the second AI, we found that the CRs in the DO and OVS groups were comparable (*p* > 0.05), and both significantly outperformed the OVS5D group (*p* < 0.05). Notably, when evaluating primiparous cows at the first AI, no significant differences were detected across all three groups (*p* > 0.05). However, at the second AI, the DO group demonstrated a significantly higher CR (*p* < 0.05) compared to both the OVS and OVS5D groups, while OVS also outperformed OVS5D (*p* < 0.05). Among multiparous cows, the CR at the first AI showed a substantial increase in the DO group compared to the OVS and OVS5D groups, which revealed similar results (*p* < 0.05). At the second AI, both primiparous and multiparous cows in the OVS and DO groups exhibited higher CRs than those in the OVS5D group (*p* < 0.05). Finally, when considering the total AI services, for both primiparous and multiparous cows, the DO group achieved the highest CRs (*p* < 0.05) compared to the OVS and OVS5D groups, which showed comparable results (*p* > 0.05). No significant differences in pregnancy loss or culling rates were detected among the three groups (*p* > 0.05), indicating similar overall results in these parameters.

**Table 1:** Independent variables and their effects on dependent variable across groups (n = 2593).

Treatment Groups	OVS (*n* = 904)	OVS5D (*n* = 830)	DO (*n* = 859)
**Independent variable (Mean ± SD)**			
Parity	1.9 ± 0.8	1.8 ± 0.9	1.9 ± 0.8
DIM	88 ± 7.8	92 ± 7.7	90 ± 8.4
Milk yield (kg)	46 ± 6.6	48 ± 5.4	49 ± 6.8
BCS	3.3 ± 0.2	3.4 ± 0.3	3.3 ± 0.1
Average days open	116 ± 10.1	134 ± 9.8	120 ± 11.2
**Dependent variable**			
No. of pregnant cows	429	384	456
No. of open cows	475	446	403
CR at 1st AI (%)	47.46 ^b^	46.27 ^b^	53.08 ^a^
aCL (%)	8.30 ^c^	65.66 ^a^	11.53 ^b^
CR at 2nd AI (%)	44.97 ^a^	35.67 ^b^	45.18 ^a^
**Primiparous cows**	*n* = 383	*n* = 206	*n* = 202
CR 1st AI (%)	54.32	57.77	55.45
CR 2nd AI (%)	47.85 ^b^	40 ^c^	62 ^a^
Total CR (%)	49.60 ^b^	45.80 ^b^	54.87 ^a^
**Multiparous cows**	*n* = 521	*n* = 624	*n* = 657
CR 1st AI (%)	42.64 ^b^	42.46 ^b^	52.64 ^a^
CR 2nd AI (%)	43.31 ^a^	34.44 ^b^	40.22 ^a^
Total CR (%)	38.61 ^b^	37.07 ^b^	43.26 ^a^
**Other Reproductive Parameters**			
Pregnancy loss (%)	5.50	6.10	6.80
Culling rate (%)	13.90	13.20	15.50

^a–c^ Values within the same row with different superscripts differ significantly (*p* < 0.05). Values for independent variables are presented as mean ± SD.

The analysis of the interaction between the FTAI protocol and embryo loss revealed a significant impact on CR at the first AI, as illustrated in Table 2. Cows in the DO group had significantly lower odds of non-conception at the first AI compared to the OVS group (*p* = 0.009). No significant difference was observed between the OVS5D and OVS groups (*p* = 0.31). Further assessment of CR at the second AI, as presented in Table 3, revealed noteworthy findings regarding the OVS5D group. Cows in this group were found to be 1.47 times more likely to encounter pregnancy loss compared to the OVS group (*p* = 0.002). In contrast, no significant difference was detected between the DO and OVS groups (*p* = 0.47). GnRH administration on day 5 post-AI is specifically intended to induce ovulation of a subordinate follicle and the formation of an aCL. Therefore, this parameter was compared across all three groups both to confirm the biological effect of this intervention in the OVS5D group and to establish the baseline rate of spontaneous aCL formation in the OVS and DO groups, which did not receive this deliberate treatment. Moreover, the evaluation of aCL formation (Table 4), showed that the OVS5D group exhibited a higher rate of aCL formation compared to the OVS group (*p* < 0.001). The DO group also showed a slightly higher rate compared to OVS (*p* = 0.01). This observation indicates that the studied FTAI protocols may have varying effects on reproductive outcomes in terms of pregnancy maintenance and aCL development.

Seasonal analysis revealed a distinct interaction between the synchronization protocol and environmental conditions, as illustrated in Table 5. During spring, OVS5D yielded the highest CR at 1st AI (*p* < 0.05), but suffered a significant reduction at 2nd AI compared to OVS group (*p* < 0.05). A critical drop in CR was observed during summer for the OVS5D group, which exhibited the lowest performance across 1st AI, 2nd AI, and total AIs (*p* < 0.05). In contrast, the OVS and DO groups showed comparable CRs during the summer months (*p* > 0.05). Autumn conditions favored the DO group, which achieved a higher CR at 1st and total AIs compared to both OVS and OVS5D groups (*p* < 0.05). In winter, DO group maintained its high CR compared to OVS5D at 1st and total AIs (*p* < 0.05).

Evaluating temporal trends within groups, OVS5D was severely impaired by summer heat stress, dropping from spring to summer (*p* < 0.05). Although DO also experienced a seasonal decline during summer, it maintained constant CR during autumn and winter, confirming its higher stability in high-yielding cows.

Additionally, to better evaluate the tested FTAI protocol, we assessed the CRs for primiparous and multiparous cows from each group (Table 6 and Table 7). Thus, for high-yielding primiparous cows (Table 6), the synchronization protocols exhibited variations primarily during extreme weather seasons. In spring and autumn, no significant differences (*p* > 0.05) were observed among groups. However, during summer, a unique divergence occurred: OVS5D achieved a significantly higher CR at 1st AI compared to OVS and DO (*p* < 0.05). This advantage was completely lost at 2nd AI, where OVS5D experienced a significant decrease compared to OVS and DO groups, which maintained comparable CRs (*p* < 0.05). In winter, OVS and DO groups presented comparable CRs and were superior compared to OVS5D at 1st AI (*p* < 0.05). Furthermore, DO group achieved an outstanding CR at 2nd AI, significantly higher than both groups (*p* < 0.05). Seasonal analysis of calving rates for primiparous cows revealed that while the OVS and DO groups remained stable across seasons, the OVS5D group was highly vulnerable during the summer months (*p* < 0.05).

In stark contrast, high-yielding multiparous cows (Table 7) revealed a critical and uniform CR pattern. During spring, OVS5D excelled at 1st AI, but significantly faded at 2nd AI compared to OVS (*p* < 0.05). Under summer season, the OVS5D group yielded significantly lower CRs at 1st, 2nd, and total AIs compared to OVS and DO groups (*p* < 0.05). Autumn environmental conditions clearly favored the DO group, which emerged as the optimal regimen for multiparous cows, yielding a superior CR at 1st and total AIs (*p* < 0.05). This trend was maintained during Winter, where DO group secured significantly higher CR at 2nd and total AIs (*p* < 0.05). Temporal analysis within the groups revealed that multiparous cows in the OVS5D group are extremely vulnerable during the summer season, as the overall CR dropped significantly from a peak in spring to a low in summer (*p* < 0.05). Conversely, the DO group demonstrated superior CR, quickly rebounding to peak efficiency during the autumn and winter season (*p* < 0.05).

**Table 6:** Seasonal variation in CRs for high-yielding primiparous cows.

Group	1 st AI	2 nd AI	Total AIs
**Spring**			
OVS	52 ^xy^ (39/75)	55.56 (20/36)	52.7 (78/148)
OVS5D	64.58 (31/48)	41.67 (5/12)	54.02 (47/87)
DO	62.79 ^x^ (27/43)	63.16 (12/19)	58.82 (50/85)
**Summer**			
OVS	47.83 ^b,y^ (44/92)	48.78 ^a^ (20/41)	45.71 (80/175)
OVS5D	61.54 ^a^ (32/52)	12.5 ^b^ (3/24)	39.81 (41/103)
DO	43.64 ^b,y^ (24/55)	56.67 ^a^ (17/30)	51.52 (51/99)
**Autumn**			
OVS	52.85 ^xy^ (65/123)	41.67 (25/60)	48.13 (116/241)
OVS5D	62.96 (34/54)	46.67 (14/30)	50 (67/134)
DO	58.49 ^xy^ (31/53)	54.17 (13/24)	57.45 (54/94)
**Winter**			
OVS	65.95 ^a,x^ (62/94)	48.98 ^b^ (24/49)	57.67 (109/189)
OVS5D	46.15 ^b^ (24/52)	51.72 ^b^ (15/29)	48.43 (62/128)
DO	52.94 ^a,xy^ (27/51)	77.78 ^a^ (21/27)	54.46 (61/112)

^a,b,^ Superscripts indicate significant differences between groups within each season (*p* < 0.05); ^x,y^ Superscripts indicate significant differences between seasons within each group (*p* < 0.05). Note: Total AIs’ represents the total number of insemination events performed per group and season, including the first and second AI, as well as any subsequent AI attempts.

**Table 7:** Seasonal variation in CRs for high-yielding multiparous cows.

Group	1 st AI	2 nd AI	Total AIs
**Spring**			
OVS	43.36 ^b^ (49/113)	53.62 ^a,x^ (37/69)	45.34 ^x^ (112/247)
OVS5D	55.4 ^a,x^ (77/139)	35.29 ^b,xy^ (24/68)	47.74 ^x^ (137/287)
DO	50.83 ^ab,xy^ (92/181)	40.21 ^b,xy^ (37/92)	44.6 ^x^ (161/361)
**Summer**			
OVS	40.45 ^a^ (36/89)	39.02 ^a,xy^ (16/41)	36.26 ^a,y^ (62/182)
OVS5D	23.52 ^b,z^ (32/136)	23.68 ^b,y^ (18/76)	22.07 ^b,z^ (66/299)
DO	46.09 ^a,y^ (59/128)	34.51 ^a,y^ (29/84)	36.73 ^a,y^ (119/324)
**Autumn**			
OVS	38.61 ^b^ (61/158)	37.5 ^y^ (36/96)	36.23 ^b,y^ (129/356)
OVS5D	43.01 ^b,y^ (77/179)	35.96 ^xy^ (41/114)	38.08 ^b,y^ (171/449)
DO	59.26 ^a,x^ (112/189)	36 ^y^ (27/75)	49.45 ^a,x^ (180/364)
**Winter**			
OVS	47.5 (76/160)	43.16 ^b,xy^ (41/95)	42.9 ^b,xy^ (160/373)
OVS5D	47.06 ^xy^ (80/170)	40.38 ^b,x^ (42/104)	42.97 ^b,xy^ (165/384)
DO	54.72 ^xy^ (87/159)	51.76 ^a,x^ (44/85)	50.44 ^a,x^ (171/339)

^a,b,^ Superscripts indicate significant differences between groups within each season (*p* < 0.05); ^x,y,z^ Superscripts indicate significant differences between seasons within each group (*p* < 0.05). Note: Total AIs’ represents the total number of insemination events performed per group and season, including the first and second AI, as well as any subsequent AI attempts.

The economic analysis tool indicated that the NPV per cow per year was higher in the DO group (EUR 35/cow/year) and lower in the OVS5D group (EUR −11.8/cow/year) compared to the OVS control group (Table 8). The profitability of the DO group increased primarily due to the improvement in the income over feed costs. This investigation assessed the economic benefits of improving CRs in high-yielding dairy cows raised in a temperate continental climate by implementing the DO protocol for the first postpartum AI. Using this reproductive strategy yielded a total net value of approximately EUR 5727.62 per cow annually. In comparison, the alternative OVS and OVS5D protocols, offered lower total net values of EUR 5692.62 and EUR 5680.82 per cow per year, respectively, as illustrated in Table 8. By using the DO protocol for the initial postpartum AI, farmers can achieve an increase in profit of approximately EUR 35/cow/year compared to the OVS protocol, and EUR 46.8/cow/per year compared to the OVS5D protocol. The findings indicate the financial implications associated with the selection of optimal reproductive strategies for dairy herds.

A sensitivity analysis was performed to test the robustness of the economic results to variation in hormone cost (Table 9) and milk price (Table 10). Across a ±20% range in milk price, the DO advantage over OVS remained positive throughout (EUR 22.43 to EUR 47.14/cow/year), and the OVS5D disadvantage remained negative throughout (EUR −10.62 to EUR −12.83/cow/year). Varying hormone dose price by the same range altered the DO advantage by no more than EUR 2.52/cow/year and the OVS5D disadvantage by no more than EUR 1.00/cow/year, in both cases without changing the sign of the effect. The economic ranking of the three protocols is therefore robust to realistic fluctuations in these two key cost parameters.

## 4. Discussion

This study evaluated the economic implications of implementing three different FTAI protocols—the traditional OVS protocol, a modified OVS protocol incorporating GnRH administration on day 5 post-AI (OVS5D), and the DO protocol— on a high-yielding dairy farm in a temperate continental climate.

The primary hypothesis of this study was that CR in high-yielding dairy cows can be significantly improved by using the OVS5D protocol, through GnRH administration on day 5 post-TAI, or the DO protocol. This approach aimed to address the challenges associated with low fertility rates in high-yielding dairy cows. By strategically timing the GnRH injection or implementing two consecutive OVS protocols, a more synchronized hormonal response was expected, potentially enhancing ovarian function and increasing the likelihood of successful conception.

In the present study, the CRs following the studied FTAI protocols were comparable between the OVS5D and OVS groups. The DO group, however, exhibited a significantly higher CR at the first AI (53.08%), compared to the OVS (47.46%) and OVS5D (46.27%) groups. The CR achieved by the DO group was higher than the results reported by Li et al. [42], which indicated a CR of 48.2% on a high-yielding dairy farm in China. Additionally, among multiparous cows, the DO protocol achieved a significantly higher CR of 52.64%, whereas the OVS and OVS5D groups showed lower rates of 42.64% and 42.46%, respectively. Primiparous cows showed comparable CRs at 1st AI across all three groups: 54.32% (OVS), 57.77% (OVS5D), and 55.45% (DO) (*p* > 0.05). At the second AI, the CRs in the DO and OVS groups were comparable (*p* > 0.05) and significantly higher than those in the OVS5D group.

A key finding of this study is that the DO protocol significantly improved fertility traits specifically in multiparous cows. This difference based on parity is closely linked to postpartum ovarian physiology. Multiparous dairy cows experience considerable metabolic demands due to their higher milk production, which may accelerate the liver’s clearance of progesterone and estradiol [43]. This could possibly explain their higher incidence of anovulation and anovulatory follicles after calving compared to the primiparous cows. The intensive double-synchronization structure of the DO protocol may therefore serve as an effective presynchronization tool. By inducing ovulation and initiating the luteal phase before the final OVS, the DO protocol likely encourages cyclicity in multiparous cows, possibly ensuring optimal progesterone levels during follicular development and enhancing the ovulation of viable oocytes, thereby explaining the significant increase in CRs observed in DO group [22,44].

In contrast, primiparous cows experienced comparable CRs across all three treatment groups, indicating that the DO protocol offers no additional reproductive advantage for this category. Primiparous cows possess a more robust ovarian follicle reserve and maintain superior, more stable postpartum endocrine profiles. They experience lower hepatic clearance of reproductive steroids because of their lower dry matter intake and milk yield compared with multiparous cows [45].

In a meta-analysis conducted by Borchardt et al. [46] it was indicated that CRs improved in primiparous cows treated with the DO protocol; however, no differences were observed in multiparous cows. Conversely, other studies found that the benefits of the DO protocol were not observed in either primiparous or multiparous cows [47]. Additionally, some reports indicated a decrease in CRs among primiparous cows subjected to the DO protocol compared to those on the conventional Presynch–Ovsynch protocol [47]. Consequently, determining which protocol is more beneficial for dairy farms remains challenging. The findings of this study indicate that for multiparous high-yielding dairy cows, the DO protocol is the most suitable option to optimize reproductive outcomes. Conversely, for primiparous cows, either the OVS protocol or the OVS5D protocol proved to be effective and sufficient for successful breeding management. Ultimately, for the second AI, cows that had been subjected to the DO protocol during the first AI showed similar CRs to those that underwent the OVS protocol. Notably, cows that received a supplementary dose of GnRH on day 5 post-AI experienced a decline in CR.

Failure to improve CRs is consistent with the results of another study in which dairy cows were treated on day 5 after AI [48]. Results of a meta-analysis [49] in which cows were treated with GnRH from 11 to 14 days after AI showed that fertility was improved in only 5 of 19 studies. However, when the analysis was limited to 6 studies having 5 common variables, GnRH treatment improved CRs. We can speculate that the aCL formed after ovulation induced by GnRH may not be fully functional [50,51]. Timing of treatment after estrus may be a critical component of the potential for GnRH to improve conception potential. Several mechanisms may explain this paradox. GnRH-induced luteal activity on day 5 post-AI alters circulating immune cell populations independently of its progesterone-elevating effect [52], and inflammatory/oxidative stress markers are independently linked to early embryonic loss in dairy cows [53]. Consistent with this, a large meta-analysis found no significant fertility benefit from GnRH-induced aCL formation, unlike hCG-induced aCL [54]. The extra injection may also add metabolic burden in high-producing cows already prone to accelerated steroid clearance [45]. Ultimately, these results highlight the importance of tailoring reproductive strategies based on parity status, shifting the paradigm toward targeted reproductive management.

The second hypothesis of this research was that an intensive FTAI protocol, such as the DO protocol, could maintain CRs during the summer months at levels comparable to those observed in other seasons. However, the data indicated that none of the studied protocols—OVS, OVS5D, or DO—maintained CRs during summer at levels comparable to those recorded in cooler seasons. Specifically, the OVS group experienced lower CRs during the summer, followed by a stabilizing plateau into autumn, rather than an immediate recovery. In contrast, the DO protocol yielded the most favorable CRs during summer and a quick rebound during autumn, whereas the lowest CRs were consistently observed in the OVS5D protocol. This clear failure of OVS5D suggests a significantly reduced efficacy under summer conditions, despite the use of a standard cooling system.

Our findings demonstrate a clear interaction between synchronization protocols, parity, and seasonal variations on the CRs of high-yielding dairy cows. The most significant finding was the decrease in CR on the OVS5D group during the summer months, particularly in multiparous cows. Although the addition of a GnRH on day 5 after AI is theoretically designed to enhance luteal function, our data suggest that under severe heat stress, this intensive hormonal manipulation becomes counterproductive in multiparous high-yielding dairy cows. Conversely, high-yielding primiparous cows under the OVS5D regimen exhibited a unique physiological resilience at first service during summer, achieving a superior CR of 61.54%. This parity-specific divergence likely stems from the lower metabolic heat production and better follicular environment of primiparous cows [55]. However, their subsequent collapse at second service highlights a latent carry-over effect of the summer season on subsequent follicular waves. On the other hand, the DO protocol demonstrated reproductive stability during autumn and winter, securing CRs around 50–51% in both primiparous and multiparous cows. The pre-synchronization phase inherent to the DO protocol effectively optimizes the physiological environment of the dominant follicle and ensures a highly functional corpus luteum at the time of timed-AI [17,22,44].

The significant drop in CRs observed during the summer occurred even though the farm was equipped with a standard cooling system. In modern high-yielding dairy farms, relying solely on convective cooling (moving air) and sprinkling is often insufficient during extreme heat stress events. When ambient temperatures exceed the cow’s upper critical limit, fans alone cannot adequately reduce the core body temperature. As a result, the follicles and subsequent embryonic development become vulnerable to thermal damage. These findings confirm that fertility is sensitive to environmental stressors [56,57]. Elevated environmental temperatures induce hyperthermia and heat stress, negatively affecting cow fertility through various mechanisms [58,59]. Both the synchronization protocol and ambient temperature can therefore influence fertility; however, the interaction between these protocols and heat stress remains poorly understood. Additionally, factors such as high milk production, mastitis, metritis, lameness, and hepatic lipolysis that influence reproductive outcomes during this period cannot be excluded [60].

A key limitation of this study is the unequal distribution of primiparous cows across the three treatment groups (42.4% in OVS, 24.8% in OVS5D, and 23.5% in DO), resulting from non-random sequential allocation based on farm management routines rather than a randomized design. Although reproductive outcomes were subsequently stratified by parity (Table 6 and Table 7) to reduce this confounding, this stratification was applied after data collection rather than built into the original allocation design, and therefore cannot fully rule out unmeasured factors correlated with both parity and treatment-group assignment. As these findings derive from a single high-yielding Holstein farm with a specific genetic background, cooling system, and management routine, extrapolation to farms with different climate, genetics, or management practices should be made with caution. Another limitation of this study is that all cows were managed on a single farm under identical housing and cooling conditions, meaning that the effects of environmental management could not be separated from those of the synchronization protocols. The seasonal differences reported here therefore reflect the performance of each protocol under one specific cooling regimen. However, this design removed between-farm variation and isolated the comparison of the three FTAI protocols. Understanding the relationship between the cooling system’s effectiveness, milk production level, and reproductive performance is critical for optimizing management practices and ensuring optimal outcomes in high-yielding dairy herds. Because all non-pregnant cows were resynchronised using the same standard OVS protocol regardless of their original treatment group, a carryover effect of the initial protocol on second-AI outcomes cannot be fully excluded, and second-AI comparisons should be interpreted considering this. The economic outputs are also based on cost and price parameters specific to the Romanian market at the time of analysis and may not be directly generalizable to other economic contexts.

Research indicates that pregnancy outcomes in cows may improve significantly when pre-synchronization is implemented using the DO protocol, as demonstrated by Souza et al. [22]. This improvement has been largely attributed to the induction of estrus cyclicity, as suggested by Gümen et al. [11] and Herlihy et al. [44]. Furthermore, an additional contributing factor is the regulation of progesterone concentration during the induction of cyclicity. The administration of GnRH during the pre-synchronization phase may facilitate more stable progesterone levels, which are essential for the establishment and maintenance of pregnancy, as supported by Herlihy et al. [44] and Vasconcelos et al. [9]. In this study, cows subjected to the DO and OVS protocols during summer exhibited a significantly higher likelihood of achieving pregnancy compared to those that received the OVS5D protocol, highlighting the reduced efficacy of the latter under severe heat stress conditions. During summer, elevated body temperatures can negatively affect fertility through multiple physiological mechanisms, including the disruption of behavioral estrus, impairment of follicular growth, and inhibition of normal ovulation [61]. A key factor contributing to estrus cyclicity failure in dairy cows involves endocrine abnormalities in plasma concentrations of FSH and inhibin. According to Sammad et al. [58], hormonal abnormalities can lead to significant reproductive challenges. These challenges begin with oocyte development, impacting fertilization competence, the estrus cycle and behavior, embryo development and implantation, the uterine environment, and even the fetal calf. Notably, GnRH administration may play a beneficial role in rectifying FSH and inhibin secretion, thereby aiding in the restoration of regular estrus cyclicity, particularly under conditions of the summer season. Furthermore, heat stress has been shown to impair CL functionality, leading to decreased progesterone levels, which are critical for successful conception [62].

Although our study focused on evaluating OVS, OVS5D, and DO protocols, existing literature suggests that modifying these protocols with exogenous progesterone supplementation could offer a heat-resilient alternative. This supplementation helps maintain elevated circulating progesterone levels during follicular development, preventing premature ovulation and the formation of subfunctional corpora lutea caused by heat stress [21]. Relying on synchronization protocol intensity alone will not offset summer fertility losses in standard-cooled, temperate continental herds. Complementary measures—shifting AI to cooler hours, upgraded evaporative cooling, and prioritizing OVS or DO over OVS5D in summer—should be prioritized in future farm management strategies.

Low CRs among high-yielding dairy cows present a significant challenge for dairy operations, resulting in considerable financial consequences [2,26]. Accordingly, the third hypothesis of this study posited that overall economic outcomes in high-yielding dairy farms could be improved by implementing either a modified OVS protocol with a GnRH agonist administered on day 5 post-AI, or the DO protocol. This strategy aimed to enhance reproductive efficiency by refining the standard synchronization protocol or by employing more intensive methods, thereby increasing the likelihood of successful conception in cows with high milk production levels. By optimizing the timing and methods of hormone administration, we expected to positively influence both productivity and profitability in the dairy cattle sector. Research by Cabrera [26,63], along with additional studies by Cabrera and Fricke [64], underlines that low CRs not only affect short-term milk production capabilities but also exert lasting effects on herd fertility, management expenses, and overall farm profitability.

In the present study, the contribution to net value was evaluated by categorizing income into several components: feed expenses, replacement costs, reproductive program costs, and calf value. Notably, incorporating a single dose of GnRH on day 5 post-AI within the OVS5D protocol resulted in an economic loss of EUR 11.8 per cow per year compared to using the OVS protocol alone. Conversely, the DO protocol yielded a net profit of approximately EUR 35 per cow per year compared with the OVS protocol and EUR 46.8 per cow per year compared with the OVS5D protocol, a benefit driven primarily by improvements in income over feed cost. Although the UW-Cornell DairyRepro$ decision tool primarily functions as a reproductive management system, its key economic metric captures how enhanced reproductive efficiency transforms herd composition. Improved CRs shorten the average calving interval, increasing the proportion of cows in early and peak lactation at any given moment. Because feed cost per cow per day changes relatively little across lactation stages while milk yield does not, the same feed input generates more milk at herd level. The higher income over feed cost reported for the DO group therefore reflects this shift in herd composition rather than any change in diet or feeding management.

The outcomes of this study align with the findings presented by Ricci et al. [19], who modeled a commercial dairy herd of 1000 cows with continuous calving, using the UW-Cornell DairyRepro$ decision tool [29]. In their model, the DO program achieved a profit of USD 42.2 (EUR 35.9) more per cow annually compared to the Presynch–Ovsynch program, accounting for hormone costs in the US market. Additionally, the DO program proved more lucrative than the 100% FTAI following a Presynch–Ovsynch program, with a profit increase ranging from USD 20.9 (EUR 17.75) to USD 44.3 (EUR 37.63) per cow annually. This economic advantage can be attributed to the higher CRs achieved by the DO protocol, which reduce the number of cows requiring re-enrollment into resynchronization programs [35]. A conventional OVS or OVS5D program requires 2 or 3 GnRH and 2 PGF2α treatments, whereas the DO program requires 4 GnRH and 3 PGF2α treatments, leading to a greater ratio of GnRH treatments compared to PGF2α. Consequently, more intensive reproductive protocols involving additional hormonal treatments that yield significantly higher CRs are more profitable than less intensive alternatives. Incorporating 7 hormonal administration in the DO protocol into herd management presents a significant trade-off between reproductive performance and practical feasibility. While the DO protocol offers better fertility outcomes and more precise synchronization compared to other methods, its complex 7-step schedule introduces specific welfare and operational challenges [65].

The findings from this study can be replicated or adapted using the UW-Cornell DairyRepro$ decision support tool, which is readily accessible to the public on the official websites of the University of Wisconsin-Madison and Cornell University. This resource enables users, researchers, and practitioners to apply the study’s findings across various dairy reproduction scenarios, making it a valuable tool for evidence-based reproductive management decisions. Although fluctuations in hormonal costs can happen across European regions, the economic conclusions of this study are still highly reliable because the model was based entirely on actual, localized farm expenses.

## 5. Conclusions

In this study, the DO protocol represents the most effective FTAI protocol for high-yielding dairy cows from a temperate continental climate, particularly when implemented from the first AI postpartum, as it significantly enhances CRs and contributes to overall farm profitability.

A key finding of this study is the necessity of tailoring reproductive management based on parity. The DO protocol is recommended for multiparous cows, which exhibited a superior reproductive response. Conversely, for primiparous cows, the standard OVS protocol alone is sufficient and effective, yielding comparable reproductive outcomes without the need for the additional hormonal interventions associated with the DO protocol. While both protocols yield comparable reproductive results in primiparous cows, the DO protocol remains preferable for maximizing reproductive efficiency in multiparous cows.

Furthermore, modifying the OVS protocol by administering a single GnRH dose on day 5 post-AI (OVS5D) failed to improve CR and resulted in a negative economic return (EUR −11.8/cow/year versus OVS). Within the conditions of the present study, this protocol did not demonstrate a reproductive or economic advantage for high-yielding dairy cows, regardless of parity or season. All three FTAI protocols yielded substantially reduced CRs during summer, confirming that standard cooling systems are insufficient to fully mitigate heat stress in herds producing around 14,000 kg/cow/year. Farms should anticipate seasonal fertility losses and prioritize parity-adapted synchronization strategies to optimize annual reproductive and economic outcomes. These results are based on a single high-yielding herd and should be applied to other farms or climates with appropriate caution. Consequently, parity-adapted synchronization remains the primary tool for optimizing reproductive and economic outcomes in standard-cooled, high-yielding dairy farms in a temperate continental climate.

## Figures and Tables

**Figure 1:**
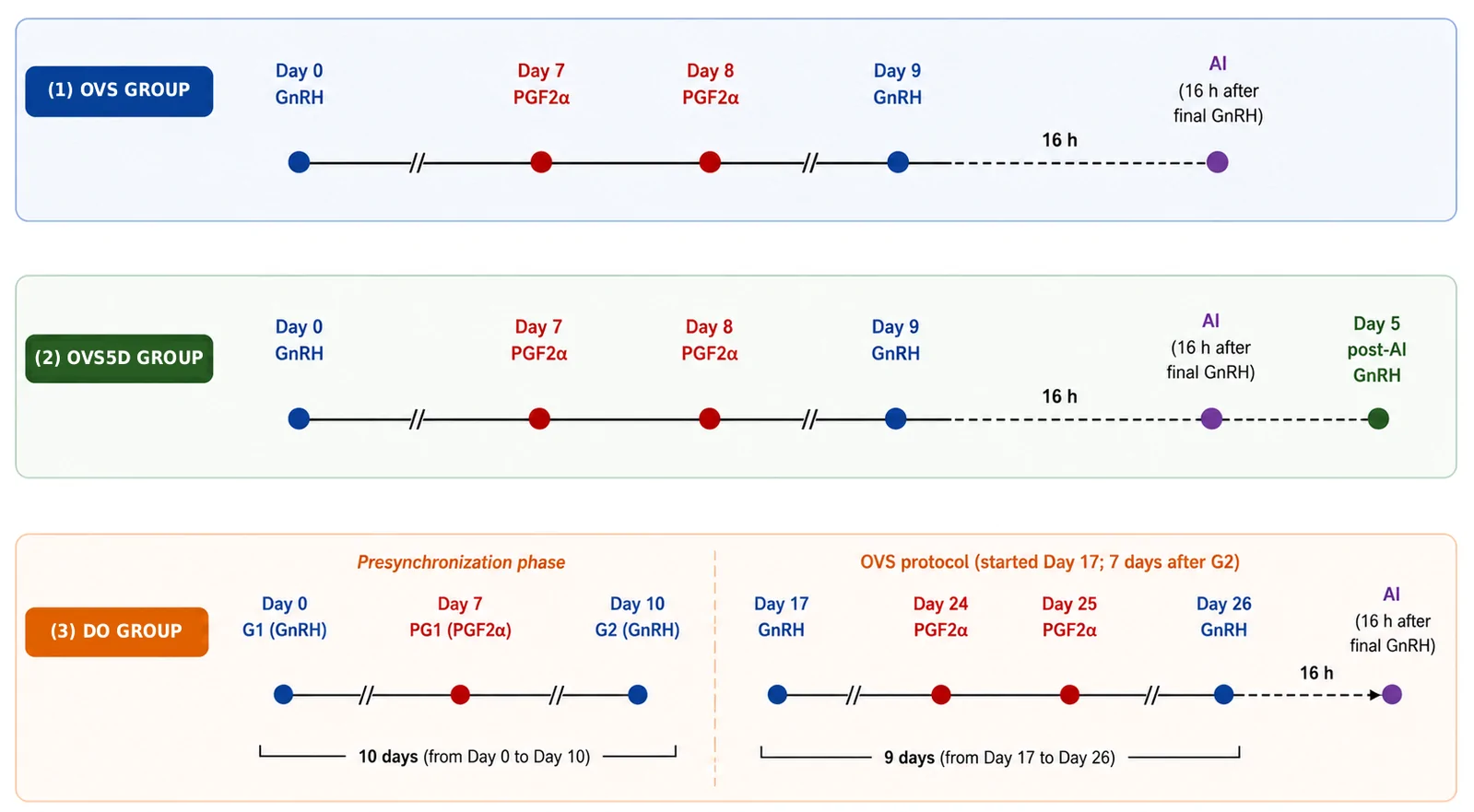
Schematic representation of the FTAI protocol’s application for each group.

**Table 2:** Odds ratios for CR at the first AI in the final logistic regression model.

Factor	Class	n	CR (%)	OR	95% CI	*p*-Value
FTAI protocol	OVS	429/904	47.46	Ref.	—	—
	OVS5D	384/830	46.27	1.05	0.87–1.27	0.31
	DO	456/859	53.08	0.80	0.66–0.96	0.009

Notes: n = number of cows that conceived/total number of cows receiving the first AI, for each protocol group; CR = conception rate; OR = odds ratio; CI = confidence interval; Ref = reference group. The model was fitted using all 2593 cows enrolled in the study. *R*^2^ Nagelkerke = 0.14.

**Table 3:** Odds ratios for CR at the second AI in the final logistic regression model.

Factor	Class	n	CR (%)	OR	95% CI	*p*-Value
FTAI protocol	OVS	219/487	44.97	Ref.	—	—
	OVS5D	163/457	35.67	1.47	1.13–1.91	0.002
	DO	197/436	45.18	0.99	0.76–1.29	0.47

Notes: n = number of cows that conceived/total number of cows receiving a second AI, for each protocol group; CR = conception rate; OR = odds ratio; CI = confidence interval; Ref = reference group. The model was fitted using the 1380 cows that received a second AI. *R*^2^ Nagelkerke = 0.12.

**Table 4:** Odds ratios for accessory Corpus luteum (aCL) formation in the final logistic regression model.

Factor	Class	n	aCL (%)	OR	95% CI	*p*-Value
FTAI protocol	OVS	75/904	8.30	Ref.	—	—
	OVS5D	545/830	65.66	0.05	0.04–0.06	<0.001
	DO	99/859	11.53	0.69	0.51–0.95	0.01

Notes: n = number of cows with aCL formation/total number of cows, for each protocol group; aCL = accessory corpus luteum; OR = odds ratio; CI = confidence interval; Ref = reference group. The model was fitted using all 2593 cows enrolled in the study. *R*^2^ Nagelkerke = 0.13.

**Table 5:** Seasonal variation in CRs for high-yielding dairy cows.

Group	1 st AI	2 nd AI	Total AIs
**Spring**			
OVS	46.81 ^b^ (88/188)	54.29 ^a,x^ (57/105)	48.10 ^x^ (190/395)
OVS5D	57.75 ^a,x^ (108/187)	36.25 ^b,x^ (29/80)	49.20 ^x^ (184/374)
DO	53.13 ^ab,x^ (119/224)	44.14 ^ab^ (49/111)	47.31 ^x^ (211/446)
**Summer**			
OVS	44.19 ^a^ (80/181)	43.90 ^a,xy^ (36/82)	40.89 ^a,y^ (146/357)
OVS5D	34.04 ^b,z^ (64/188)	21 ^b,y^ (21/100)	26.61 ^b,z^ (107/402)
DO	45.36 ^a,y^ (83/183)	40.35 ^a^ (46/114)	40.19 ^a,y^ (170/423)
**Autumn**			
OVS	44.84 ^b^ (126/281)	39.10 ^y^ (61/156)	41.04 ^b,y^ (245/597)
OVS5D	47.64 ^b,y^ (111/233)	38.19 ^x^ (55/144)	40.82 ^b,y^ (238/583)
DO	59.09 ^a,x^ (143/242)	40.4 (40/99)	51.09 ^a,x^ (234/458)
**Winter**			
OVS	54.33 ^a^ (138/254)	45.13 ^xy^ (65/144)	47.86 ^ab,xy^ (269/562)
OVS5D	46.85 ^b,y^ (104/222)	42.86 ^x^ (57/133)	44.34 ^b,xy^ (227/512)
DO	54.29 ^a,x^ (114/210)	58.03 (65/112)	51.44 ^a,x^ (232/451)

^a,b,^ Superscripts indicate significant differences between groups within each season (*p* < 0.05); ^x,y,z^ Superscripts indicate significant differences between seasons within each group (*p* < 0.05). Note: Total AIs’ represents the total number of insemination events performed per group and season, including the first and second AI, as well as any subsequent AI attempts.

**Table 8:** Economic analysis and contribution to net value (EUR/cow/year).

Items	OVS Group	OVS5D Group	DO Group	Diff. OVS5D-OVS	Diff. DO-OVS	Diff. DO-OVS5D
Income over feed cost	5798.12	5792.77	5858.43	−5.35	60.31	65.66
Calf value	97.34	96.41	95.65	−0.93	−1.69	−0.76
Replacement cost	−162.75	−164.11	−176.17	−1.36	−13.42	−12.06
Reproductive cost	−40.09	−44.26	−50.29	−4.16	−10.19	−6.03
Total net value	5692.62	5680.82	5727.62	−11.80	35	46.8

Notes: EUR = Euro; Diff. = difference relative to the OVS reference group, except for the Diff. DO−OVS5D column, which shows the direct difference between the DO and OVS5D groups. All economic values are expressed in EUR per cow per year. Negative values represent costs or lower returns compared to the reference group. Positive values indicate a financial advantage over the OVS group.

**Table 9:** Hormone price sensitivity analysis.

Variation vs. Baseline	GnRH (EUR/Dose)	PGF2α (EUR/Dose)	Diff. OVS5D−OVS (EUR/Cow)	Diff. DO−OVS (EUR/Cow)
−20%	4.00	2.08	−10.80	37.52
−10%	4.50	2.34	−11.30	36.26
Baseline	5.00	2.60	−11.80	35.00
+10%	5.50	2.86	−12.30	33.74
+20%	6.00	3.12	−12.80	32.48

Notes: EUR = Euro; Diff. = difference in total net value relative to the OVS reference group, recalculated from the Table 8 baseline using the indicated hormone price. Negative values represent a lower return relative to OVS; positive values indicate a financial advantage over OVS. Baseline hormone prices: GnRH = EUR 5.00/dose, PGF2α = EUR 2.60/dose. Across the full ±20% range tested for both parameters, the Diff. OVS5D−OVS values remained negative throughout and the Diff. DO−OVS values remained positive throughout, indicating the economic ranking of the three protocols is robust to realistic variation in hormone dose price.

**Table 10:** Milk price sensitivity analysis.

Variation vs. Baseline	Milk Price ($/cwt)	Diff. OVS5D−OVS (EUR/Cow)	Diff. DO−OVS (EUR/Cow)
−20%	22.94	−10.62	22.43
−10%	25.80	−11.22	28.59
Baseline	28.67	−11.80	35.00
+10%	31.54	−12.32	41.07
+20%	34.40	−12.83	47.14

Notes: EUR = Euro; Diff. = difference in total net value relative to the OVS reference group, recalculated from the Table 8 baseline using the indicated milk price. Negative values represent a lower return relative to OVS; positive values indicate a financial advantage over OVS. Baseline milk price: EUR 0.50/kg (USD 28.67/cwt). Across the full ±20% range tested for both parameters, the Diff. OVS5D−OVS values remained negative throughout and the Diff. DO−OVS values remained positive throughout, indicating the economic ranking of the three protocols is robust to realistic variation in milk price.

## Data Availability

The data presented in this study are available on request from the corresponding author due to privacy restrictions.

## Abbreviations

The following abbreviations are used in this manuscript:

AI	Artificial insemination
CL	Corpus luteum
FTAI	Fixed timed artificial insemination
DO	Double Ovsynch
OVS	Ovsynch
OVS5D	Ovsynch combined with GnRH administration on day 5 post timed artificial insemination
THI	Temperature-humidity index
DIM	Days in milk
VWP	Voluntary waiting period
NPV	Net present value
DM	Dry matter
OR	Odds ratio
CI	Confidence interval
BCS	Body condition score
SD	Standard deviation
CR	Conception rate
aCL	Accessory corpus luteum
